# Sacral and Pelvic Insufficiency Fractures Following Adult Spinal Deformity Surgery: A Case Report and Systematic Literature Review

**DOI:** 10.3390/jcm14217572

**Published:** 2025-10-25

**Authors:** Calogero Velluto, Achille Marciano, Gianmarco Vavalle, Maria Ilaria Borruto, Andrea Perna, Laura Scaramuzzo, Luca Proietti

**Affiliations:** 1 Department of Aging, Orthopaedic and Rheumatological Sciences, Fondazione Policlinico Universitario Agostino Gemelli IRCCS, Largo A. Gemelli, 8, 00168 Rome, Italy; calogero.velluto@guest.policlinicogemelli.it (C.V.); achille.marciano01@icatt.it (A.M.); gianmarco.vavalle01@icatt.it (G.V.); mariailaria.borruto01@icatt.it (M.I.B.); luca.proietti@policlinicogemelli.it (L.P.); 2 Department of Orthopaedics and Traumatology, Fondazione Casa Sollievo della Sofferenza IRCCS, 71013 San Giovanni Rotondo, Italy; perna.andrea90@gmail.com

**Keywords:** sacral insufficiency fracture, pelvic insufficiency fracture, adult spinal deformity, spinal fusion, spinopelvic fixation, osteoporosis

## Abstract

**Background**: Sacral and pelvic insufficiency fractures (SIFs and PIFs) are increasingly recognized yet frequently underdiagnosed complications after adult spinal deformity (ASD) surgery, particularly in patients undergoing long-segment spinal fusion to the sacrum or pelvis. **Methods**: We present a representative case of sacral and pelvic insufficiency fractures following extensive spinal fusion, highlighting diagnostic and therapeutic challenges. In addition, a systematic review of the literature was performed according to PRISMA guidelines through PubMed, MEDLINE, and Scopus databases, including studies up to December 2024. Data regarding demographics, risk factors, diagnostic modalities, management strategies, and outcomes were extracted and narratively synthesized. **Results**: A total of 21 studies comprising 89 patients were included. The majority were elderly postmenopausal women with osteoporosis and additional risk factors such as chronic corticosteroid therapy or high body mass index. Diagnosis was frequently delayed due to low sensitivity of plain radiographs, whereas computed tomography was the most reliable modality. Management was surgical in 49 patients (55%)—most commonly extension of fixation to the pelvis or use of S2-alar-iliac screws—with favorable fracture healing reported in most cases. Conservative treatment, employed in 40 patients (45%), included bracing, restricted activity, and bone health optimization, also leading to healing in the majority of cases. **Conclusions**: Sacral and pelvic insufficiency fractures represent an underrecognized but clinically significant complication after ASD surgery. Early recognition through cross-sectional imaging (CT/MRI) is crucial, and both surgical and conservative approaches can be effective if tailored to patient and fracture characteristics.

## 1. Introduction

Sacral and pelvic insufficiency fractures (SIFs and PIFs) are increasingly recognized yet often underdiagnosed complications following adult spinal deformity (ASD) surgery, especially in patients undergoing long-segment instrumented fusions to the sacrum or pelvis. Although significant advancements in surgical correction of complex spinal deformities have been achieved in recent decades, these procedures inevitably determine alteration of the biomechanics of the lumbosacral junction and sacropelvic region, increasing the risk for stress-related fractures in susceptible patients, such as osteoporotic patients [1,2].

Due to their often insidious onset, presenting as low back or sacral pain, these fractures can easily be misattributed to routine postoperative discomfort or degenerative disease, leading to diagnostic delays. Plain radiographs have a low sensitivity for detecting sacral insufficiency fractures—some series report diagnostic accuracy as low as 31%—whereas advanced cross-sectional imaging such as computed tomography (CT) or magnetic resonance imaging (MRI) is required for definitive diagnosis [3,4].

The altered load distribution after long spinal fusions creates abnormal stress concentration at the sacropelvic junction, particularly in constructs with significant sagittal imbalance, high pelvic incidence-lumbar lordosis mismatch, or pseudarthrosis at the lumbosacral level [4]. As biomechanical analyses have demonstrated, spinopelvic fixation can effectively redistribute these forces and reduce the risk of progressive instability, yet the decision to extend fusion constructs to the ilium must balance the benefits against increased operative time, blood loss, and implant-related complications [2,4].

When unrecognized, sacral and pelvic insufficiency fractures can have a deep impact on patient outcomes, potentially resulting in persistent pain, functional decline, progressive deformity, or implant failure [5]. Although conservative management—including bracing, activity modification, and optimization of bone health—may be effective in selected cases, a substantial proportion of patients ultimately require surgical intervention with lumbopelvic fixation to achieve pain relief and prevent further complications [1,3]. However, the current literature remains limited, consisting predominantly of single-case reports and small series, underscoring the need for greater awareness and consensus on diagnostic and therapeutic algorithms.

This study therefore presents a representative case illustrating the diagnostic challenges and management considerations of sacral and pelvic insufficiency fractures after ASD surgery, supported by a systematic review of the literature to summarize current evidence on risk factors, diagnostic approaches, and outcomes. Unlike prior systematic reviews that addressed sacral insufficiency fractures in heterogeneous or mixed postoperative populations, this study specifically focuses on sacral and pelvic insufficiency fractures occurring after adult spinal deformity (ASD) surgery. By consolidating these data, this work aims to inform clinical decision-making and encourage early recognition of this frequently underestimated complication.

## 2. Materials and Methods

### 2.1. Search Strategy

**Figure 1:**
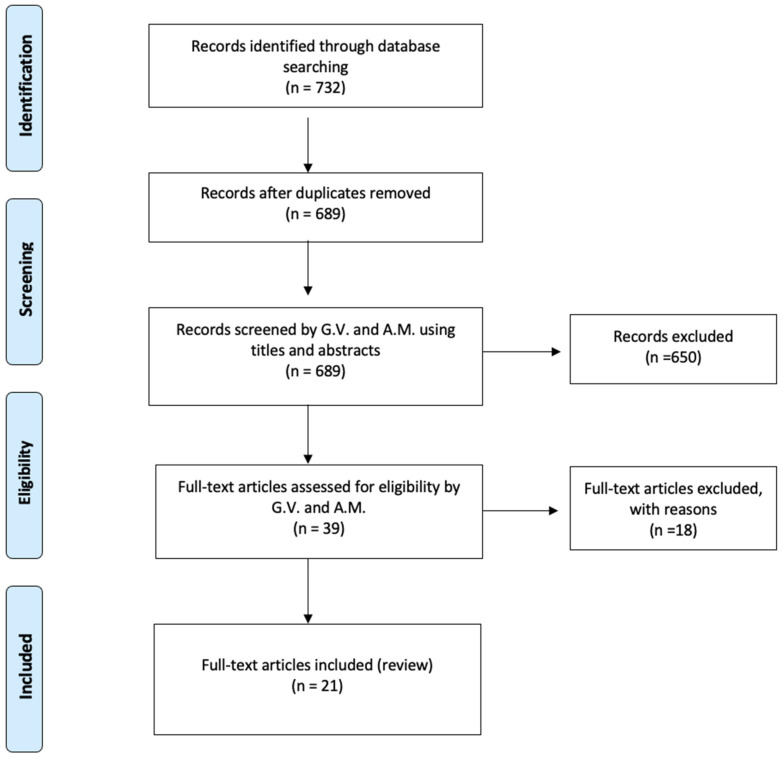
PRISMA flowchart.

This systematic review was conducted according to the Preferred Reporting Items for Systematic Reviews and Meta-Analyses (PRISMA) guidelines as showed in Figure 1. A comprehensive literature search was performed using PubMed, MEDLINE, and Scopus databases to identify relevant studies published up to December 2024. The following search terms were used in various combinations with Boolean operators “AND” and “OR”: “sacral fracture”, “pelvic insufficiency fracture”, “adult spinal deformity”, “spinal fusion”, “spinopelvic fixation”. Medical Subject Headings (MeSH) were included where applicable. Reference lists of selected articles and relevant reviews were also screened to identify additional studies. The search was reiterated to ensure inclusion of the most up-to-date evidence.

### 2.2. Inclusion and Exclusion Criteria

Eligible articles for this review included case reports, case series, and retrospective or prospective studies reporting demographic characteristics, risk factors, clinical presentation, diagnostic modalities, treatment strategies, and outcomes of sacral and pelvic insufficiency fractures following adult spinal deformity surgery. Only full-text articles in English were considered. Studies focused on fractures unrelated to prior spinal instrumentation, those addressing only surgical techniques without clinical data, animal studies, cadaveric investigations, editorials, expert opinions, and conference abstracts were excluded. Duplicates were removed during the screening process.

### 2.3. Data Collection

Two authors (G.V. and A.M.) independently screened titles and abstracts for relevance. Full texts of potentially eligible studies were subsequently reviewed to confirm inclusion criteria were met. Any discrepancies were resolved through consensus with a third reviewer (C.V). Extracted data included: patient demographics, type and extent of spinal instrumentation, risk factors (e.g., osteoporosis, steroid use), timing and modality of diagnosis, fracture classification when available, management approach (conservative vs. surgical), and clinical outcomes. Data were cross-verified to ensure accuracy and consistency with the original reports. Missing data (e.g., BMI, T-scores) were reported as ‘Not available’ and excluded from quantitative synthesis, but retained qualitatively for completeness.

### 2.4. Statistical Analysis

Data were organized using Microsoft Excel (Microsoft Corporation, Redmond, WA, USA) for tabulation and descriptive statistical analysis. Categorical variables were summarized as frequencies and percentages, while continuous variables were reported as means and standard deviations where applicable. Due to the heterogeneity of included studies and the predominance of individual case reports and small series, a meta-analysis was not feasible. Instead, narrative synthesis and tabular presentation were employed to illustrate key findings and facilitate comparison across reported cases. Study screening and data extraction were performed independently by two reviewers. Inter-rater agreement was high (Cohen’s κ = 0.87).

## 3. Case Report

A 72-year-old woman initially presented in early 2010 with chronic low back pain and bilateral radiculopathy in an L5–S1 distribution, associated with neurogenic claudication. Symptoms were exacerbated by standing and walking, and relieved by sitting or lumbar flexion. Radiographic and MRI studies demonstrated a low-grade isthmic spondylolisthesis at L5–S1 with foraminal stenosis.

In December 2010, she underwent posterior decompression and L5–S1 arthrodesis with pedicle screws and rods. Initial postoperative recovery was uneventful, but symptoms progressively recurred over the following two years. Imaging revealed adjacent segment degeneration, and in April 2012, she underwent a second surgery with posterior extension of the fusion to L3 (L3–S1 fixation) due to junctional failure and worsening alignment.

In November 2014, she underwent a third intervention with extreme lateral interbody fusion (XLIF) at L3–L4 and L4–L5 to improve anterior column support, restore disk height, and reinforce the construct, which had become increasingly unstable.

**Figure 2:**
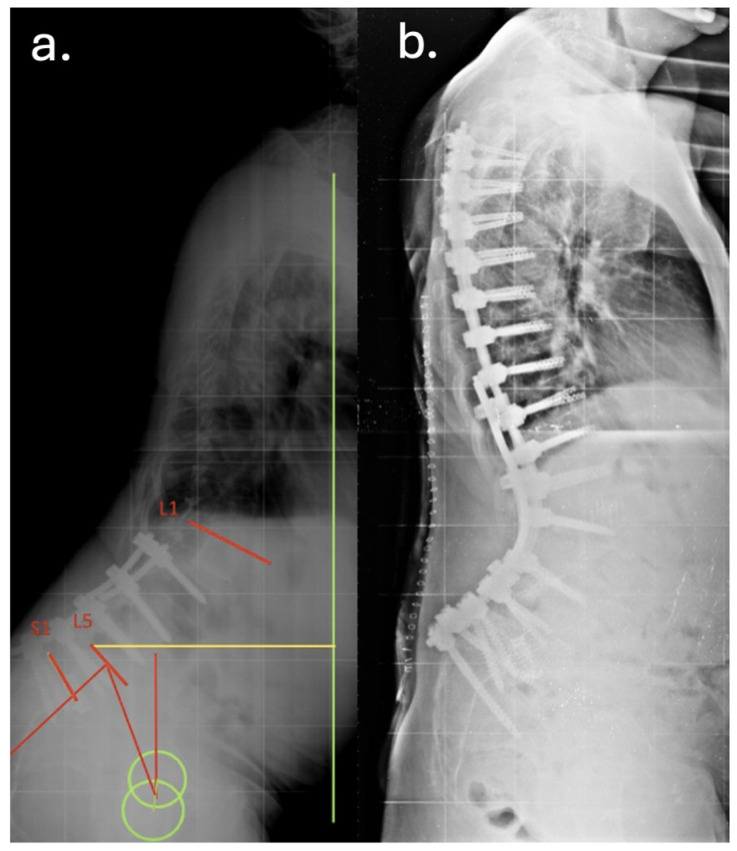
Preoperative (**a**) and postoperative (**b**) lateral radiographs documenting the first revision surgery with extension of the arthrodesis from T2 to the ilium. The images show restoration of sagittal alignment, with correction of the sagittal vertical axis (SVA) and improvement of lumbar lordosis.

**Figure 3:**
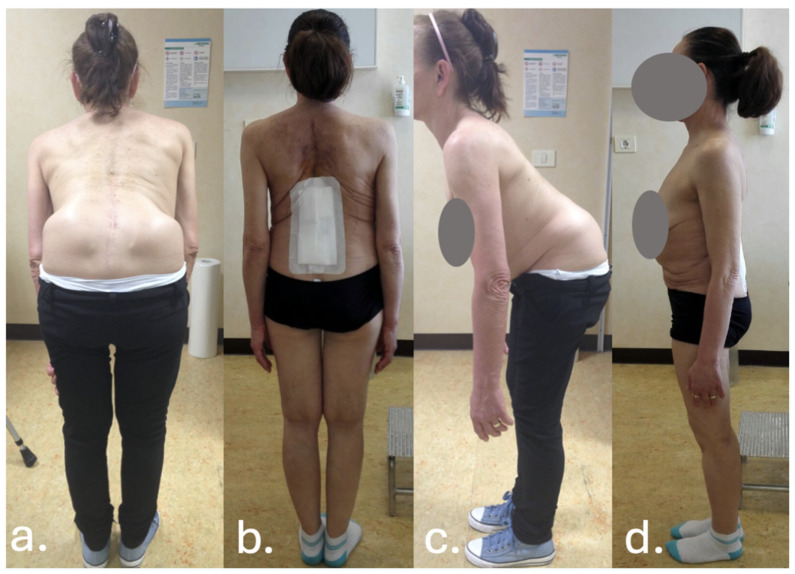
Clinical photographs of the patient before (**a**,**c**) and after (**b**,**d**) revision surgery. Preoperative images (**a**,**c**) demonstrate coronal trunk shift and severe sagittal imbalance with marked forward trunk flexion. Postoperative images (**b**,**d**) show improvement of overall posture, with correction of coronal alignment and restoration of sagittal balance following extension of fusion from T2 to the ilium.

Despite temporary clinical improvement, progressive sagittal imbalance and worsening pain prompted a fourth surgery in June 2016, she underwent a major posterior revision: all prior instrumentation was removed and replaced with new rods and screws extending from T7 to the pelvis, including bilateral iliac fixation as showed in Figure 2 and Figure 3. Postoperatively, the patient developed high-grade fever and was diagnosed with a urinary tract infection due to *Acinetobacter baumannii XDR*. She was treated with multiple antibiotic courses, including teicoplanin, tigecycline, and colistin.

Between August and September 2016, and again in March and April 2017, she required four further surgical revisions due to persistent wound dehiscence and local signs of infection, though systemic markers remained intermittently elevated.

**Figure 4:**
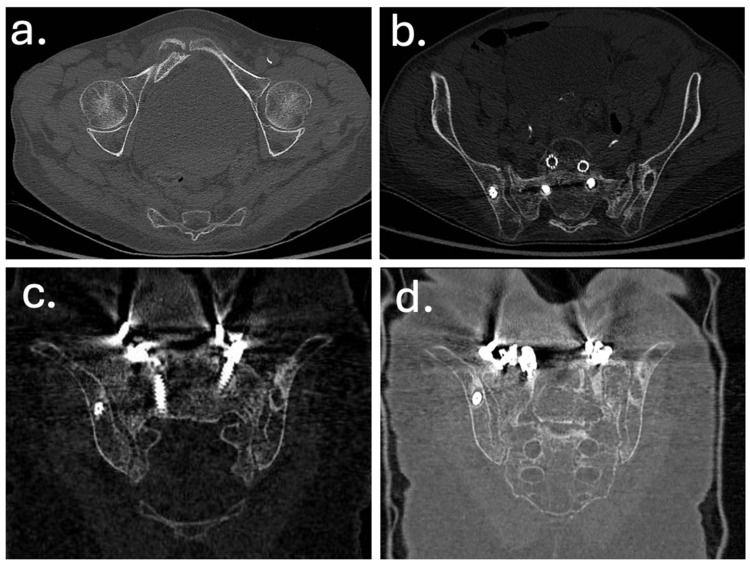
Computed tomography (CT) images obtained at the time of sacral insufficiency fracture diagnosis. (**a**) Axial view showing a right ilio-pubic branch fracture. (**b**) Axial view demonstrating an impacted fracture of the left sacral ala. (**c**,**d**) Coronal reconstructions confirming the left sacral ala fracture (**c**) and illustrating loosening of the iliac screws (**d**).

**Figure 5:**
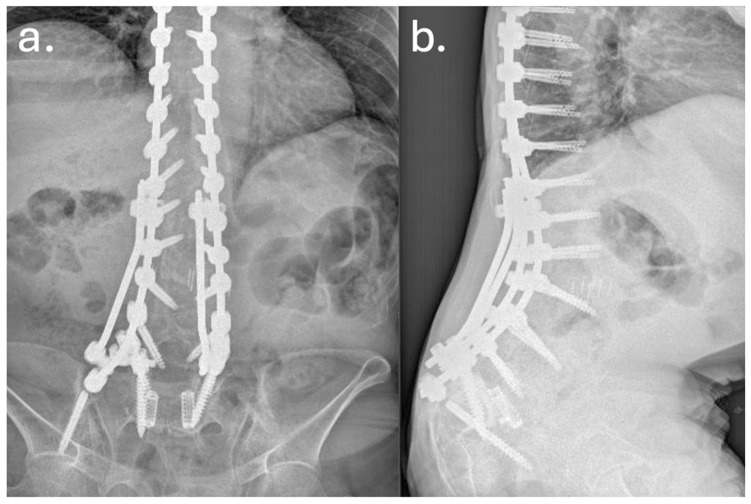
Anteroposterior (**a**) and lateral (**b**) radiographs at 2-year follow-up. The images demonstrate loosening of the bilateral iliac screws with peri-implant radiolucency, consistent with mechanical failure at the lumbopelvic junction. The sagittal view shows partial loss of the achieved lumbar lordosis and persistent sagittal imbalance, suggesting progressive biomechanical compromise of the construct despite maintenance of overall instrumentation continuity.

In December 2019, a revision surgery was performed due to partial construct failure and suspected iliac screw loosening as showed in Figure 4 and Figure 5. The left iliac screw and bilateral screws at T4–T5 were removed. New pedicle screws were placed bilaterally at T2–T3, and two additional rods were added, creating a delta-rod four-rod configuration to enhance construct stability and distribute stress.

In January 2020, a rotational musculocutaneous flap was performed by the plastic surgery team for soft tissue reinforcement and coverage.

In 2022, the patient was admitted to another hospital with fever and abdominal pain. Lumbar spine MRI showed hyperemia and bone marrow edema of the right sacral ala and iliac joint surface, adjacent to a displaced screw, raising suspicion of chronic deep infection.

In September 2022, surgical debridement was performed. A cutaneous fistula tract was excised, the protruding rod was shortened, and copious irrigation was completed. A V-Y fasciocutaneous flap was advanced from the left flank to close the soft tissue defect. Intraoperative microbiological swabs were obtained.

In late 2024, a new fistula appeared in the lumbar region. In March 2025, intraoperative revision revealed mobility and fracture of the four proximal thoracic screws. The rods were cut proximally, and all four fractured screws were removed. Deep tissue biopsies and purulent fluid samples were collected. All four intraoperative cultures grew *Staphylococcus epidermidis*, while one was additionally positive for *Ralstonia pickettii*. A fasciocutaneous flap was again used to manage the soft tissue defect.

At her most recent evaluation (May 2025), the patient was afebrile and in stable condition. The flap appeared well healed, without signs of dehiscence or drainage. She denied significant pain. Laboratory tests including white cell count, CRP, liver and renal function were all within normal limits. Full-spine radiographs confirmed persistent sagittal and coronal imbalance, but the remaining hardware was unchanged and stable. Despite mechanical compromise and reduced autonomy—she ambulated short distances with a crutch and relied on a wheelchair for longer distances—no further revision was indicated due to the high surgical risk. Suppressive antibiotic therapy with amoxicillin/clavulanate 875/125 mg BID was continued, with scheduled laboratory monitoring and follow-up. A chronological summary of the patient’s surgical procedures, infectious complications, and sacral insufficiency fracture diagnosis is provided in Table 1.

## 4. Literature Review

### 4.1. Demographical Data

A total of 21 studies were included, reporting 89 patients affected by sacral and pelvic insufficiency fractures (SIFs and PIFs) following adult spinal deformity (ASD) surgery, as showed in Table 2. The mean age across the reported cases was 64.6 years (range 48–73 years), with a clear predominance of elderly, postmenopausal women (72 females, 17 males). This aligns with the well-known higher prevalence of osteoporosis and reduced bone mineral density in this demographic.

Most patients underwent instrumented spinal fusion for ASD correction, degenerative scoliosis, fixed sagittal imbalance, or high-grade spondylolisthesis. The mean length of fusion constructs was 4 vertebral levels, although constructs ranged from short-segment lumbar fusions (L4–S1) to long thoracolumbosacral constructs extending to the pelvis or including iliac screws. For example, Buell et al. described a series of 9 patients who developed sacral insufficiency fractures after multilevel lumbosacral arthrodesis, highlighting the mechanical vulnerability of the sacropelvic junction when spinopelvic fixation is omitted [4]. Similarly, Holderread et al. reported 7 patients who developed pelvic stress fractures following decompression and multilevel instrumentation for spinal stenosis [6], while Yasuda et al. described 8 cases of sacral and pubic fractures among 53 ASD patients, emphasizing the difference in risk between constructs with and without iliac screws [7]. Klineberg et al. contributed with a cohort of 9 patients with degenerative L5/S1 spondylolisthesis treated with various lumbosacral constructs, where junctional fractures were identified as a cause of persistent pain and hardware failure [2]. Bone health status was often suboptimal. Where reported, T-scores frequently fell below −2.5, consistent with severe osteoporosis. Several patients had additional risk factors, including chronic corticosteroid therapy, rheumatoid arthritis or systemic lupus erythematosus, obesity, and metabolic bone disease. In some series, such as that of Pennekamp et al., patients developed sacral stress fractures after short-segment fusion to S1, suggesting that even limited constructs can generate pathological stress distribution when bone quality is compromised [8]. Timing of diagnosis varied considerably. The interval from index surgery to fracture detection ranged from as early as 2 weeks to over 12 months, with a median diagnostic delay of several months in many series.

### 4.2. Risk Factors and Pathophysiology

Across the 21 studies included in this review, various patient-related and procedure-related risk factors were described in association with sacral and pelvic insufficiency fractures after adult spinal deformity surgery. Advanced age and female sex predominated; most patients were elderly, postmenopausal women with reduced bone mineral density. Several studies explicitly reported T-scores below −2.5. For example, Buell et al. described severe osteoporosis in multiple patients who sustained sacral stress fractures following long instrumented fusion [4]. Similarly, Ganeshan et al. and Holderread et al. reported cases where bone mineral density was documented by DEXA scans [6,9]. Chronic corticosteroid therapy was noted in several case reports, including those by Koh et al., Pennekamp et al. and Fourney et al., describing patients with rheumatoid arthritis or systemic lupus erythematosus who required long-term immunosuppressive treatments [8,10,11]. In the series by Papadopoulos et al., five patients within a larger ASD cohort developed sacral insufficiency fractures [12]; multiple patients in this subset had prolonged steroid exposure or additional metabolic risk factors. Obesity was reported as an additional modifying factor in some series. For example, Kolz et al. described overweight patients who sustained fractures within months of surgery, and Ha et al. noted that several cases involved patients with coexisting diabetes mellitus and high BMI [13,14]. Scemama et al. similarly reported patients with elevated BMI undergoing multilevel fusions who developed sacral stress fractures in the first postoperative year [15]. Long fusion constructs extending to S1 without spinopelvic fixation were highlighted as a relevant biomechanical contributor. Yasuda et al. found a higher rate of sacral and pubic fractures in patients treated without iliac screws [7], while Klineberg et al. described fractures occurring below fusions ending at the sacrum [2]. Elias et al. and Mathews et al. included patients who had short or moderate lumbar constructs but still developed stress fractures, suggesting that even limited fusions can generate abnormal stress if bone quality is poor [16,17]. Buell et al. described pseudarthrosis at the lumbosacral junction in several cases, which may create abnormal micromotion and junctional stress [4]. In Noh et al., a high-grade spondylolisthesis was treated with L4–S1 fusion, and a sacral fracture was diagnosed months later after progressive new-onset pain [18]. The timing of fracture diagnosis varied among the included studies. In Bose et al., a patient with prior spondylolysis developed a sacral fracture within weeks of surgery, while other series reported detection up to one year postoperatively [19]. Although sacral insufficiency fractures were the most common, some studies, such as Yasuda et al., also described associated pubic rami fractures or ischiopubic stress fractures occurring simultaneously [7]. Where described, additional factors such as prior pelvic radiotherapy and metabolic bone disorders were reported in specific cases. For example, Vavken et al. included patients with a history of pelvic irradiation for gynecologic malignancy, and Mathews et al. mentioned patients with multiple prior vertebral fractures and evidence of metabolic bone disease beyond osteoporosis [17,20].

### 4.3. Diagnostic Workup and Imaging

Diagnostic assessment was inconsistently reported across the included cases, but where specified, cross-sectional imaging was the most frequently used modality for confirming sacral or pelvic insufficiency fractures. Among the 89 patients analyzed in this review, CT scanning was performed in 14 cases (15.7%), making it the most common diagnostic tool. Plain radiographs were reported in 5 patients (5.6%), either as an initial study or as part of routine postoperative follow-up, but these were often described as insufficient for definitive diagnosis due to the subtle nature of stress fractures. MRI was used in 2 patients (2.2%), mainly to detect early bone marrow edema and to exclude other postoperative complications such as infection or tumor. Scintigraphy or SPECT imaging was not specifically reported in the included cases, although other systematic reviews, such as the one by Meredith et al., have highlighted its occasional role in detecting occult insufficiency fractures when CT findings are equivocal [1]. The systematic review by Ganeshan et al. [9] emphasized that conventional radiography alone has low sensitivity, with some series reporting that standard X-rays failed to detect sacral fractures in up to 50% of cases. CT, on the other hand, was consistently described as the most reliable modality for visualizing sacral ala fractures and associated pelvic ring disruptions. In the larger series reported by Buell et al., cross-sectional imaging was routinely used when patients presented with unexplained sacral or buttock pain after long spinal fusions [4]. Yasuda et al. similarly described CT as essential in identifying both sacral and ischiopubic fractures that were not visible on plain films [7]. MRI was described in a few reports, such as by Holderread et al., as helpful for detecting early stress reactions or bone marrow edema, particularly when CT was inconclusive [6]. The timing of diagnostic imaging varied considerably, reflecting the frequent delay in clinical suspicion. In several cases, including those by Noh et al. and Papadopoulos et al., patients underwent multiple imaging studies before an insufficiency fracture was confirmed [12,18]. This diagnostic delay ranged from a few weeks to several months postoperatively. Across the cases reviewed, the imaging findings were often correlated with worsening or new-onset low back, sacral, or pelvic pain not attributable to hardware complications.

### 4.4. Treatment Strategies

Among the 89 patients included in this review, treatment approaches were clearly reported for all cases. Revision surgery was performed in 49 patients (55%), while 40 patients (45%) were managed conservatively. The decision between operative and non-operative management varied according to fracture displacement, persistent pain, and failure of initial conservative care. Overall, the studies consistently describe a preference for revision surgery when fractures were unstable or when conservative measures failed to provide adequate symptom relief. For example, Buell et al. reported nine patients who all required revision surgery with extension of the fusion construct to the pelvis using bilateral iliac screws, upsizing of L5–S1 screws, or the addition of multiple accessory rods to reinforce the sacropelvic junction [4]. Similarly, Ha et al. and Kolz et al. described patients who underwent complex revision strategies such as quad iliac bolts, upsizing sacral screws, and decompression when indicated [13,14]. Scemama et al. also detailed three patients managed operatively with quad iliac bolts and upsize of existing hardware. In some cases, less invasive techniques were also described [15]. Ganeshan et al. presented a patient treated with bilateral percutaneous sacroplasty using a long-axis approach combined with bilateral ileosacral screw fixation to improve stability [9]. Papadopoulos et al. reported five patients who all underwent revision surgery; four required additional anterior lumbar interbody fusion (ALIF) for anterior column support alongside extension to the pelvis with iliac screws [12]. Revision strategies frequently involved innovative techniques tailored to each patient’s anatomy and biomechanical demands. Noh et al. reported a patient managed with direct S1–S2 fusion, while Klineberg et al. and Yasuda et al. described the use of bilateral S2 alar-iliac (S2AI) screws and quad iliac bolts, often with accessory rods for added construct strength [2,7,18]. In some reports, multiple staged procedures were necessary to address both proximal and distal junctional failures.

Conservative management, chosen in 40 patients, generally consisted of structured bed rest, external bracing such as thoracolumbosacral orthosis (TLSO), gradual rehabilitation, and pharmacological optimization of bone health where indicated. Studies such as those by Holderread et al., Mathews et al., Khan et al., Fourney et al. and Vavken et al. described cases managed successfully without surgery when fractures were non-displaced and patients could tolerate conservative protocols [6,11,17,20,21]. Koh et al. reported a patient managed with lumbar bracing and activity modification, while Bose et al. described a case that initially started conservatively but progressed to revision surgery due to nonunion and persistent pain [10,19]. Notably, some series included mixed approaches within the same cohort. For example, Holderread et al. managed four patients operatively and three conservatively, depending on the degree of instability [6]. Klineberg et al. described two patients treated with revision surgery and seven with non-operative measures such as bracing and observation [2]. Yasuda et al. reported six patients requiring S2AI screw fixation and two patients who achieved fracture healing with conservative immobilization and gradual rehabilitation over 8–16 weeks [7]. Additional single-patient reports further illustrate this range of approaches. Wang et al. detailed a case treated with decompression and extension of the construct using iliac screws [22]. Asad et al. and Elias et al. each presented patients treated, respectively, with S2AI screws and conservative pain control and water therapy [16,23]. Pennekamp et al. described a patient successfully managed with bracing and careful mobilization [8]. Finally, Ganeshan et al. and Noh et al. highlight less common techniques such as percutaneous sacroplasty and novel sacral fixation methods [9,18]. Across these reports, revision surgery most frequently involved extension to the pelvis with iliac or S2AI screws, upsizing or replacing hardware, and, when indicated, anterior support with ALIF or sacroplasty. Non-operative management generally relied on bed rest, bracing, restricted activity, and gradual return to function under careful monitoring.

### 4.5. Outcomes

Across the included studies, outcome data were reported for the majority of patients, though follow-up detail varied. Overall, 54 out of 89 patients (60%) had a clearly documented outcome indicating clinical or radiographic healing, while 2 patients (2.2%) experienced complications requiring further interventions. For a small minority of cases, outcome details were not explicitly stated. Buell et al. reported nine patients with complete radiographic fusion confirmed by X-rays between 12 and 18 months postoperatively [4]. Kolz et al. described six patients who achieved sacral fracture healing within six to twelve months on follow-up CT and radiographs [13]. Scemama et al. similarly reported three patients with sacral consolidation confirmed on CT scans at six, eight, and twelve months, respectively [15]. In single-patient reports, Ganeshan et al. observed partial consolidation at ten months [9], while Fourney et al. documented complete healing on radiographs at nine months [11]. Wang et al., Noh et al., Papadopoulos et al., Bose et al., Pennekamp et al., Elias et al. each described clinical healing and symptomatic improvement [8,12,16,18,19,22]. The cohort by Holderread et al. provided more specific details: four of seven patients were ambulating independently without symptoms at follow-ups ranging from nine to twenty months, while two developed complications such as surgical site infection and discitis, requiring further revision surgery [6]. Klineberg et al. reported nine patients who achieved clinical healing with significant symptomatic improvement following revision procedures [2]. Yasuda et al. described eight patients who showed complete fracture consolidation and symptom resolution after immobilization for eight to sixteen weeks [7]. Importantly, Mathews et al. reported three patients who healed completely within six to twelve months [17] while Khan et al. documented full healing at an average of eight months for all three patients [21]. For a few reports, including Vavken et al., Koh et al. and Asad et al., specific outcome details were not provided in the available data [10,20,23].

**Table 2:** Demographic data of patients in the included studies of the systematic literature review.

Author	Year	N° Patients	Sex	Age	Surgical Treatment	Stress Fracture	Timing	Diagnostic Imaging	T Score	Treatment	Outcome
Velluto et al. [this paper]	2025	1	F	65	T4-Pelvis	Sacral and Ischiopubic fractures	1 year after surgery	CT	T < −3.1	Operative revision surgery: removed iliac screws, flap for exposure of screws and bed rest	Restoration and osteoporosis therapy
Ganeshan et al. [9]	2023	1	F	73	T4–S1 ciao	Sacral insufficiency fracture	1.5 month after surgery	CT	-	Operative. Bilateral percutaneous sacroplasty with spinal cement + bilateral percutaneous ileosacral fixation	Restoration
Holderread et al. [6]	2022	7	2 (M) and 5 (F)	-	-	-	-	-	-	3 non operative/4 operative	-
			F	78	L2–L5	Bilateral L5 pedicle fractures, L5–S1 fracture with spondylolisthesis, L5–S1 discoligamentous injury	2 wk	CT/MRI	-	Operative: L5–S1 posterior SF with instrumentation, autogenous BG and allograft bone morcellized	Ambulating with walker, no symptoms after 14 months
			F	71	L2–S1	-	2 wk	CT	-	Operative: L3 pelvic fusion with S1/S2 Lam	-
			F	67	L3–S1	S1–S2 subluxation with sacral fracture below level of previous fusion	4 wk	CT/MRI	-	Operative: L3–S1 posterior SF with instrumentation and allograft bone, L5–S1 revision Lam, autogenous BG	Surgical site infection, chronic L2 compression fracture after 4 months
			F	77	L4–S1	S1 sacral insufficiency fracture below hardware	2 wk	CT/MRI	-	Nonoperative: Lumbar bracing	Restoration after 20 months
			F	67	L2–S1	Sacral fracture with early loosening of inferior pedicle screws	8 mo	CT/MRI	-	Operative: L4–S1 removal of hardware, L3–L4 revision with discectomy, revision L2–S1 posterolateral SF with instrumentation, application of incisional wound vac	C. albicans T12–L2 discitis/osteomyelitis with epidural abscess 10 mo postoperatively; another revision surgery (T12–L1 Lam, T8–L3 fusion, T12, L1 corpectomy) (1 yr)
			M	71	L3–S1	Transverse sacral ala fracture involving central body and bilateral ala	3 wk	XR/CT	-	Nonoperative: Bone stimulator, lumbar bracing	Ambulating with walker, no symptoms (9 mo)
			M	79	L4–S1	L5–S1 anterolisthesis	3 mo	CT	-	Nonoperative: Standard postoperative rehabilitation with observation	Ambulating without assistance (1 yr)
Buell et al. [4]	2020	9	4 (M) and 5 (F)	73 ± 6 (mean)	-	-	-	-	-	-	-
			M	80	L2–S1 PSIF, PCO (L2–S1), TLIF (L5–S1)	S1 peri-screw lucency, superior S1 body Fx w/ventral implant displacement, high-grade L5–S1 anterolisthesis, rt sacral ala Fx	4 wk	CT	-	Operative. Upsize L5 screws (8.5 mm), remove S1 screws, quad iliac bolts (9.5 mm), 3rd and 4th accessory rods	Healed on CT (8 mo)
			F	79	T10–S1 PSIF	H-type sacral Fx, transverse Fx below S1 foramina	2 wk	CT	Hx of osteoporosis, DEXA hip T-score −2.4, no recent Tx	Operative. Re-instrumentation, quad iliac bolts (9.5 mm), 3rd and 4th accessory rods	Healed on XR (10 mo)
			F	64	T3–S1 PSIF, PCO (T11–L4)	H-type sacral Fx, transverse Fx below S1 screws	5.5 yr	CT	None	Operative. Quad iliac bolts (9.5 mm), 3rd and 4th accessory rods	Healed on XR (1 yr.)
			F	79	L2–S1 PSIF, PCO (L3–5)	Transverse sacral Fx involves S1 screws, lt sacral ala Fx	3 wk	CT	Hx of osteoporosis, no recent DEXA or Tx	Operative. Quad iliac bolts (9.5 mm), Grafton DBF	Healed on XR (1 yr.)
			M	70	T10–S1 PSIF, PCO (T12–L2)	Complex T-like sacral Fx, transverse Fx from rt L5–S1 disk space (prior fusion) to lt S1 screw, lt sacral ala Fx extends to lt S2–3 foramen	1 yr	CT	-	Operative. Extension to T4 (for PJK), quad iliac bolts (9.5 mm)	
			M	70	L4–S1 PSIF, TLIF (L4–5, L5–S1)	H-type sacral Fx, transverse Fx below S1 foramina, L5–S1 pseudarthrosis	3 wk	CT	Osteopenia, DEXA hip T-score −1.3, no recent Tx	Operative. Upsize L4–S1 screws, quad iliac bolts (9.5 mm superiorly, 10.5 mm inferiorly)	S1-Iliac rod Fx requiring revision surgery approximately 2 yrs postop
			M	66	L5–S1 PSIF, TLIF (L5–S1)	U-type sacral Fx, transverse Fx below S1 screws, L5–S1 pseudarthrosis	4 wk	CT	Hx of osteoporosis, DEXA hip T-score −1.0, teriparatide	Operative. Extension to L4, replace L5–S1 screws, L5–S1 cross-link, iliac bolts (9.5 mm)	Early CT, healed on XR (4 yrs)
			F	76	L4–S1 PSIF, L5 Gill laminectomy, TLIF (L5–S1)	U-type sacral Fx, transverse Fx involves S1 screws	3 wk	CT	Osteoporosis, no recent DEXA, calcitonin nasal spray	Operative. Upsize S1 screws, decompress lt L5 nerve root, quad iliac bolts (8.5 mm)	Healed on CT, (7.5 yrs)
			F	69	L4–S1 PSIF, TLIF (L4–5, L5–S1)	H-type sacral Fx, transverse Fx involves S1 screws	3 wk	CT	Steroid-induced osteoporosis, DEXA hip T-score −1.6, wrist T-score −1.5, alendronate	Operative. Upsize S1 screws, quad iliac bolts (8.5 mm, 9.5 mm)	Healed on CT (3.5 yrs)
Scemama et al. [15]	2016	3	3 (F)	72 (mean)							
			F	72	L3–S1 decompression, posterolateral fusion and instrumentation L2–S1 (bilateral S1 screws only) with one level Smith-Petersen osteotomy without iliac crest bone graft	Transversal S1 fracture with L5–S1 pseudarthrosis	2 mo	XR/CT	-	Operative two-time revision surgery, firstly posterior revision with bilateral iliac extension, secondly an anterior interbody L4–L5 and L5-S1 fusion using cage + 3 months protection brace	Healed on CT (6 mo)
			F	77	Posterior liberation posterolateral fusion and instrumentation extension to S1 (bilateral S1 pedicular screws only)	Sacral proximal plate fracture with S1 screws failure	2 wk	XR/CT	-	Operative. L5-S1 decompression, bilateral iliac extension of posterolateral fusion and instrumentation	Healed last FU XR (8 mo)
			F	67	T12–S1, bilateral iliac extension posterolateral fusion and instrumentation (modular fixation, pedicular S1 and iliac screws	U-Type sacral Fracture, with a proximal implants failure	2 mo	CT	-	Operative. Revision surgery with proximal extension to T11 and distal posterior extension with 2 iliac implants on the right side and 1 implant on the left side. Bilateral iliac crest graft and artificial bone were used. Additional anterior approach with anterior L1–L2, L2–L3, L3–L4, L4–L5 and L5–S1 cage interbody fusions + additional protection brace for three months	Sacral fracture consolidation on CT, last FU (1 yo p.o.)
Koh et al. [10]	2005	1	F	48	L4–S1 decompression and fusion; 11 revisions for pseudarthrosis, adjacent level failure, infection, and hardware failure	transverse fracture of the bilateral pelvic wing-sacrum	7 mo	XR/CT	-	Nonoperative. brace and gradual rehabilitation and pain control	-
Asad et al. [23]	2019	1	M	73	L2–S1	Sacral insufficiency fracture	2 days	XR/CT	-	Operative S2 sacral alar iliac screws to extend fixation	-
Ha et al. [14]	2021	8	F	72.13 (mean)							
			F	83	L3–S1, PLF	Bilateral vertical sacral fracture	17 yr	SPECT/CT	−2.8	Operative. Bilateral S2AI screw	-
			F	74	L3–S1, PLIF	Bilateral vertical sacral fracture	8 mo	CT	−4	Operative. Bilateral S2AI screw	-
			F	75	L5–S1, PLIF	Unilateral vertical sacral fracture	10 yr	SPECT/CT	−4.2	Nonoperative	Resolution 8–16 weeks
			F	83	L4–S1, PLIF	Bilateral vertical sacral fracture	10 yr	SPECT/CT	−2	Nonoperative	Resolution 8–16 weeks
			F	53	L1–S1, PLF	Unilateral vertical sacral fracture	3.5 yr	SPECT/CT	−3.2	Nonoperative.	Resolution 8–16 weeks
			F	54	L1–S1, PLIF	Unilateral vertical sacral fracture	6 yr	Bone scan/CT/MRI	−2.7	Nonoperative.	Resolution 8–16 weeks
			F	76	L2–S1, OLIF/PLF	Horizontal sacral fracture	1 mo	CT/MRI	−2.7	Operative. Bilateral S2AI screw	-
			F	79	T7–S1, PLF	Unilateral vertical sacral fracture	12 yr	SPECT/CT	−2.3	Operative. Pedicle subtraction osteotomy at L4 with S2AI fixation for ipsilateral SIF	-
Kolz et al. [13]	2020	6	4 M and 2 F	59.83 (mean)							
			M	60	L5–S1, 2-stage anterior–posterior fusion	fracture of the superior sacral endplate + subsidence of his anterior cage	3 wks	CT	−1.6	Operative, Bed rest for 3 weeks, then revision L4 to pelvic fusion with bilateral iliac screws	Healed at 6 months
			M	76	L5–S1, 2-stage ALIF and PSF	S1 insufficiency fracture	4 wks	CT	−2.2	Operative. L4 to pelvis revision fusion	Healed at 6 months
			M	66	L5–S1, 2-stage ALIF and PSF	S1 insufficiency fracture with cage subsidence and screw loosening	3 mo	CT	-	Operative. Initial management included rest, transforaminal epidural steroid injection but did not control pain. L4-pelvis decompression and L4–L5 transforaminal lumbar fusion with instrumentation (TLIF) and iliac screws	Healed at 1 year
			F	73	L5–S1, ALIF	S1 insufficiency fracture	17 days	CT	−1.5	Operative. L5–S2 sacral alar-iliac fusion	Healed at 2 years
			M	53	L5–S1, ALIF	S1 insufficiency fracture	1 mo	CT	-	Operative. L5–S2 sacral alar-iliac fusion	
			F	31	L5–S1, LAIF	S1 insufficiency fracture	3 mo	CT	-	Previous 3 months conservative then degeneration of the superior adjacent level with L5–S1 pseudarthrosis and subsidence of the ALIF cage. Operative. 2-stage L4–S1 anterior spinal fusion with instrumentation and posterior spinal fusion	
Mathews et al. [17]	2001	3	F	71.33 (mean)							
			F	74	L3–S1 decompression, pedicle screw fixation, and fusion with right-sided iliac crest bone graft	fracture of the sacrum with additional fractures of the right superior and inferior pubic rami	2 wks	XR	-	Nonoperative. Physical therapy (gradual), pain control	Healed on XR (8 mo)
			F	70	L1–S1 decompression, pedicle screw instrumentation, and fusion with right-sided iliac crest bone graft	displaced transverse sacral insufficiency fracture	1 mo	XR	-	Nonoperative. Bedrest followed by mobilization (gradual)	Radiographs at 8 months revealed scarla fracture healing but in a forward displaced position
			F	70		Superior pubic ramus fracture and a right iliac fracture	2 wks	XR	-	Nonoperative.	Healed 2 yr.
Khan et al. [21]	2005	3	F	64.33 (mean)							
			F	57	L4–L5 laminectomy with bilateral foraminotomies, with L4 S1 posterior spinal fusion with instrumentation and left-sided iliac crest bone graft	Junctional S2 stress fracture	2 mo	XR	-	Nonoperative Continue functional exercise with observation	Healed on XR (1 yr.)
			F	79	L3–L5 laminectomy with bilateral foraminotomies as well as L3–S1 posterior spinal fusion with instrumentation and left-sided iliac crest bone graft were performed	Junctional sacral fracture just below the S1 screws	6 wks	XR	-	Nonoperative. Instructed on level of activity, given donut pillow for sitting	Healed 14 mo
			F	57	Lumbar laminectomy with instrumented fusion from L4 to sacrum, with right posterior iliac crest bone graft	Left parasymphyseal stress fracture + sacral stress fracture below the S1 pedicle screw	6 wks	-	-	Nonoperative. Pain management and observation and water therapy	Healed 22 mo
Fourney et al. [11]	2002	1	F	61	L5 laminectomy with bilateral L5–S1 facetectomies and foraminotomies; L5–S1 pedicle screw fixation with reduction in spondylolisthesis; L5–S1 discectomy + PLIF with allograft; posterolateral fusion with allograft and autograft	sacral insufficiency fracture with anterolisthesis of S1 on S2	4 wks	XR/CT	-	Nonoperative Thoracolumbosacral orthosis for 3 months	Healed on XR (9 mo)
Vavken et al. [20]	2008	4									
			F	77	T12–S1	S2 fracture	5 wks	CT		Operative: Extension of the arthrodesis to S2 with iliac screws	Resolution
			F	65	L2–S1	S1 fracture	4 mo	CT	severe osteoporosis	Nonoperative	Resolution
			F	79	T10–S1	S1 fracture	Immediate post-op	CT		Nonoperative	Resolution
			M	78	T11–S1	S1 fracture	5 wks	CT		Nonoperative	Resolution
Wang et al. [22]	2016	1	F	65	L4–S1	Horizontal sacral fracture at the S1/S2 level with complete displacement of S2 and associated neurological deficits	5 yrs	CT	−3.7	Operative: Decompression and extension of arthrodesis with iliac screw fixation	Resolution
Noh et al. [18]	2016	1	1 F	64	L4–S1	Sacral stress fracture	5 yrs	CT	-	Operative	Resolution
Papadopoulos et al. [12]	2008	5	4 F, 1 M	67.4	Thoraco-lumbar fusion	5 Sacral stress fracture	4–6 mo	CT	-	Operative: iliac screws and ALIF	Resolution
Bose et al. [19]	2003	1	F	41	Decompression and fusion L4–S1	S1–S2 fractures	7 mo	CT	-	Non Operative: bed rest for 3 months	Resolution
Pennekamp et al. [8]	2005	1	F	57	L4–S1 fusion	Sacral stress fracture	9 days	XR and CT	-	Nonoperative	Resolution
Elias et al. [16]	2002	1	F	53	L4–S1 fusion	Sacral stress fracture	4 mo	MRI and CT	-	Nonoperative	Resolution
Klineberg et al. [2]	2008	9	F	64 (mean)	Arthrodesis from 1 to 8 segments	Two sacral insufficiency fractures in short fusions (1.3%). Four sacral insufficiency fractures (3.1%) in long fusions. Three fractures referred from other centers	5 wks	CT	-	Two operative cases: patients initially treated surgically. Seven non-operative cases: managed conservatively with bracing and analgesic therapy, four of whom discontinued treatment and subsequently underwent surgery. Surgical treatment consisted of decompression and L4–L5 laminectomy, L4–L5 arthrodesis with pedicle screws and two iliac screws per side (sacral screws placed at the surgeon’s discretion)	Resolution

## 5. Discussion

### 5.1. Pathophysiology and Diagnostic Challenges

Sacral and pelvic insufficiency fractures (SIFs and PIFs) represent a biomechanical consequence of altered load distribution at the sacropelvic junction following long-segment spinal fusion. Constructs terminating at S1 without additional spinopelvic fixation generate abnormal stress concentrations, increasing susceptibility to junctional fractures [2,14]. Advanced age, osteoporosis, chronic corticosteroid therapy, and prior pelvic irradiation further amplify this risk [4,5]. Our findings confirm that elderly, osteoporotic, postmenopausal women constitute the most vulnerable subgroup, a demographic repeatedly highlighted across multiple series [1,6]. The cumulative burden of poor bone quality, high mechanical demands, and extensive instrumentation creates a “perfect storm” for fracture development, particularly in patients with sagittal imbalance or pseudarthrosis at the lumbosacral junction [4]. Because symptoms are often nonspecific and radiographs have low sensitivity, early cross-sectional imaging—particularly CT and MRI—is pivotal for timely recognition in high-risk patients [9,14]. Delayed or missed diagnosis is one of the most critical issues in managing SIFs and PIFs. Clinical manifestations are often subtle, typically presenting as low back, sacral, or buttock pain that can be misattributed to postoperative discomfort or degenerative disease. Plain radiographs, despite their widespread use, demonstrate limited sensitivity, with diagnostic accuracies reported as low as 31% [1]. This emphasizes the necessity of maintaining a high index of suspicion in postoperative ASD patients, especially those with persistent pain or functional decline not explained by hardware complications. Preventive measures include pre-operative optimization of bone health (e.g., DXA screening, vitamin D/calcium supplementation, anti-osteoporotic therapy) and spinopelvic fixation techniques (e.g., S2-alar-iliac screws) to reduce junctional stress.

### 5.2. Management Strategies

Management of sacral and pelvic insufficiency fractures must be tailored to patient characteristics, fracture stability, and construct integrity. Approximately half of the patients in our review underwent revision surgery, which commonly involved extension of the fusion to the pelvis, iliac or S2AI screw placement, and upsizing of lumbosacral fixation [5,8]. Innovative approaches such as percutaneous sacroplasty with supplemental fixation have been reported in selected high-risk patients [6]. Conversely, conservative strategies—activity modification, bracing, and bone health optimization—remain viable in non-displaced fractures and fragile patients, with several series documenting satisfactory healing under non-operative protocols [6,10]. Importantly, Yasuda et al. (2016) demonstrated that even in elderly patients, fracture healing can be achieved within 8–16 weeks of immobilization, provided that early recognition and careful monitoring are ensured [7].

### 5.3. Clinical Implications

The dual challenges of fracture prevention and early recognition call for a multidisciplinary approach. Preoperative assessment and optimization of bone quality are paramount, particularly in elderly women with osteoporosis. Pharmacological intervention (e.g., bisphosphonates, denosumab, or anabolic agents such as teriparatide) may improve bone strength and reduce the risk of postoperative fractures. From a surgical standpoint, consideration of spinopelvic fixation in high-risk patients must balance the benefits of biomechanical reinforcement against the risks of increased operative time, blood loss, and implant-related complications [4]. Postoperatively, a structured surveillance program including advanced imaging in symptomatic patients may facilitate earlier detection and improve outcomes.

### 5.4. Limitations

This study has several limitations. First, the systematic review is constrained by the heterogeneity of available evidence, which predominantly consists of case reports and small retrospective series. This inherently reduces the strength of conclusions and increases susceptibility to publication bias, as unusual or severe cases are more likely to be reported. Second, variability in reported outcomes, imaging modalities, and follow-up protocols limited the comparability of data across studies and precluded meta-analysis. Third, the review was restricted to English-language publications, potentially excluding relevant evidence from other sources. Finally, our illustrative case report, while representative of the clinical complexity surrounding sacral insufficiency fractures, reflects a single-patient experience and therefore limits generalizability. In addition to heterogeneity and lack of risk-of-bias assessment, treatment variability across studies further limits comparability. Future multicenter prospective studies with standardized diagnostic and outcome reporting are needed to establish evidence-based guidelines and to define optimal protocols.

Moreover, no formal risk-of-bias assessment could be performed, given the methodological heterogeneity and descriptive nature of the included reports. This lack of standardized appraisal, combined with the substantial variability in patient populations, index constructs, and follow-up duration, underscores the need for prospective multicenter studies with uniform diagnostic criteria, standardized outcome measures, and longer-term follow-up to establish more definitive conclusions.

Overall, our findings align with the current literature in underscoring that sacral and pelvic insufficiency fractures following adult spinal deformity surgery are underrecognized yet clinically impactful complications. Preventive strategies focusing on bone health, surgical planning, and postoperative surveillance are essential. The literature review demonstrates that sacral and pelvic insufficiency fractures following adult spinal deformity surgery can achieve high rates of healing when managed appropriately, although a minority of patients may require additional revisions due to complications.

## 6. Conclusions

Sacral and pelvic insufficiency fractures following adult spinal deformity surgery remain an underrecognized but clinically significant complication. They predominantly affect elderly, osteoporotic women undergoing long fusion constructs, particularly those ending at the sacrum without adequate spinopelvic fixation. The insidious onset and nonspecific presentation often result in delayed diagnosis, highlighting the importance of maintaining a high index of suspicion in patients presenting with new or worsening sacropelvic pain after surgery. Advanced imaging modalities, especially CT and MRI, are essential for accurate detection, given the poor sensitivity of plain radiographs. Management strategies must be individualized, ranging from conservative measures such as bracing and bone health optimization to revision surgery with lumbopelvic fixation in cases of instability or persistent symptoms. Although outcomes are generally favorable when appropriately managed, delayed recognition may contribute to chronic pain, functional decline, and hardware failure. This case, together with the systematic review, underscores the necessity of preoperative optimization of bone quality, careful selection of fusion levels, and proper use of spinopelvic fixation in high-risk patients. Greater awareness among spine surgeons and multidisciplinary teams is crucial to facilitate timely diagnosis and to develop standardized treatment algorithms aimed at improving patient outcomes.

## Figures and Tables

**Table 1:** Chronological summary of surgical procedures, infectious complications, and the sacral insufficiency fracture diagnosis in the case report patient.

Year	Event
Dec 2010	Posterior decompression and L5–S1 fusion
Apr 2012	Posterior extension of fusion to L3 for junctional failure
Nov 2014	Extreme lateral interbody fusion (XLIF) at L3–L4 and L4–L5
Jun 2016	Major revision from T7 to pelvis with bilateral iliac fixation
Aug 2016	Postoperative infection (*Acinetobacter baumannii XDR*), multiple antibiotic courses
Dec 2019	Removal of left iliac and T4–T5 screws, new fixation at T2–T3 with 4-rod construct
Jan 2020	Rotational musculocutaneous flap for soft tissue coverage
Feb 2022	Diagnosis of sacral insufficiency fracture (CT: left sacral ala right ilio-pubic branch fracture, with loosening of iliac screws)
Sep 2022	Surgical debridement, rod shortening, V-Y fasciocutaneous flap
Mar 2025	Removal of fractured proximal thoracic screws; cultures positive for *Staphylococcus epidermidis* and *Ralstonia pickettii*
May 2025	Stable condition on suppressive antibiotics; no further revision indicated

## Data Availability

The data presented in this study are available on request from the corresponding author due to privacy.
